# DNA Methyltransferase Candidate Polymorphisms, Imprinting Methylation, and Birth Outcome

**DOI:** 10.1371/journal.pone.0068896

**Published:** 2013-07-26

**Authors:** Paul Haggarty, Gwen Hoad, Graham W. Horgan, Doris M. Campbell

**Affiliations:** 1 Lifelong Health, Rowett Institute of Nutrition and Health, University of Aberdeen, Aberdeen, United Kingdom; 2 Biomathematics and Statistics Scotland, Aberdeen, United Kingdom; 3 Department of Obstetrics and Gynaecology, University of Aberdeen, Aberdeen, United Kingdom; University of Bonn, Institut of experimental hematology and transfusion medicine, Germany

## Abstract

**Background:**

Birth weight and prematurity are important obstetric outcomes linked to lifelong health. We studied a large birth cohort to look for evidence of epigenetic involvement in birth outcomes.

**Methods:**

We investigated the association between birth weight, length, placental weight and duration of gestation and four candidate variants in 1,236 mothers and 1,073 newborns; *DNMT1* (rs2162560), *DNMT3A* (rs734693), *DNMT3B* (rs2424913) and *DNMT3L* (rs7354779). We measured methylation of LINE1 and the imprinted genes, PEG3, SNRPN, and IGF2, in cord blood.

**Results:**

The minor DNMT3L allele in the baby was associated with higher birth weight (+54 95% CI 10,99 g; p = 0.016), birth length (+0.23 95% CI 0.04,0.42 cm; p = 0.017), placental weight, (+18 95% CI 3,33 g; p = 0.017), and reduced risk of being in the lowest birth weight decile (p = 0.018) or requiring neonatal care (p = 0.039). The DNMT3B minor allele in the mother was associated with an increased risk of prematurity (p = 0.001). Placental size was related to PEG3 (p<0.001) and IGF2 (p<0.001) methylation. Birth weight was related to LINE1 and IGF2 methylation but only at p = 0.052. The risk of requiring neonatal treatment was related to LINE1 (p = 0.010) and SNRPN (p = 0.001) methylation. PEG3 methylation was influenced by baby DNMT3A genotype (p = 0.012) and LINE1 by baby 3B genotype (p = 0.044). Maternal DNMT3L genotype was related to IGF2 methylation in the cord blood but this effect was only seen in carriers of the minor frequency allele (p = 0.050).

**Conclusions:**

The results here suggest that epigenetic processes are linked birth outcome and health in early life. Our emerging understanding of the role of epigenetics in health and biological function across the lifecourse suggests that these early epigenetic events could have longer term implications.

## Introduction

Factors such as birth weight and placental weight are important indicators of the health of the newborn but they have also been linked to health and biological function in later life [1]–[3]. However, the relationships are complex with higher birth weight being associated with reduced cardiovascular mortality but a higher risk for certain cancers [2], [3] including breast cancer [4]. There are also complex links with socioeconomic position, employment status and cognition. Lower birth weight and hospitalisation during childhood are associated with lower occupational position and adverse changes in cardiometabolic factors and an increased risk of metabolic syndrome [5]. Cognitive ability is associated with birth weight [6], [7] and intelligence in youth is associated with all-cause-mortality [8]. Prematurity is also associated with long term disadvantage and increased risk of mortality throughout childhood [9].

The nature of these associations and the causal links between them are not well understood. It has been proposed that nutritional and other environmental influences in pregnancy may programme both birth weight and health in later life [1], [2]. However, there is a heritable component to birth weight and length of gestation [9]–[11] and many of the adult diseases linked to birth weight [12]. This has led to an alternative hypothesis that common genetic pathways underpin prenatal growth and adult health outcomes [3], [11], [13]. There is now a third category, based on epigenetics, which opens up the possibility that either or both explanations may be correct [14]–[16]. Knowledge of the causal mechanism is important as it could lead to different strategies to improve health across the lifecourse.

We set out to look for evidence of epigenetic involvement in birth outcomes in a large birth cohort. The role of epigenetics in human complex traits is difficult to study as the epigenome of readily accessible tissues such as blood may not reflect the epigenome within the tissues of interest. Perhaps paradoxically, evidence for the involvement of epigenetic processes may be obtained from genetic association studies. DNA methylation is probably the most commonly studied epigenetic phenomenon. We investigated the association between birth weight, length, placental weight and duration of gestation and four candidate polymorphisms in genes involved in DNA methylation in mothers and newborns. Polymorphisms were measured in the four DNA methyltransferases: DNMT1 (MIM126375); DNMT3A (MIM602769); DNMT3B (MIM602900); DNMT3L (MIM606588) in order to provide additional information on specific epigenetic process.

Recent work suggests that many imprinted genes maintain their allele specific methylation signal in a wide range of adult somatic tissues, independent of the absolute levels of expression [17]. We measured DNA methylation in the imprinted genes – Paternally expressed gene 3 (PEG3; 19q13; OMIM 601483); Insulin-like growth factor II (IGF2; 11p15; OMIM 147470); Small nuclear ribonucleoprotein polypeptide N (SNRPN; 15q12; OMIM 182279) – and the transposable element – Long interspersed nuclear element 1 (LINE-1; OMIM 151626) in newborn cord blood and determined the association between these and birth weight, length, placental weight and duration of gestation.

## Materials and Methods

The study was approved by Grampian Research Ethics Committee and all women in the study gave informed written consent to take part. Women who met the inclusion criteria were recruited consecutively. Women who were diabetic, carrying multiple pregnancies, who conceived as a result of fertility treatment, or in whom clinical data was not available, were excluded. Information on each pregnancy was abstracted from Aberdeen Maternity and Neonatal Databank and the data anonymised. Low birth weight was defined as the lowest decile for the standardised birth weight score; this is the birth weight z score adjusted for gestational age, sex and parity [18]. Deliveries before 37 weeks gestation were defined as pre-term. Pregnancies in which the newborn was admitted to the neonatal unit for any reason were categorised as requiring neonatal treatment.

### Variant Selection and Genotyping

Women provided a blood and buccal sample whilst attending for ultrasound scan in Aberdeen Maternity Hospital and a sample of cord blood was collected at delivery. The concentration of DNA was determined by TaqMan RNaseP detection assay (Applied Biosystems, Warrington, UK) before genotyping. Genotypes were detected by allelic discrimination assay using TaqMan® MGB probes labelled with 6-FAM™ and VIC® on a 7500 Fast real-time PCR system (Applied Biosystems, Warrington, UK) [19]. We used the targeted candidate gene/polymorphism approach [20] which has the advantage of minimising the number of variants studied and hence reducing the risk of false positive results. Variants (*DNMT1* MIM126375 19p13.3-p13.2; rs2162560; *DNMT3A* MIM 602769; 2p23; rs734693; *DNMT3B* MIM602900; 20q11.2; rs2424913; *DNMT3L* MIM606588; 21q22.3; rs7354779) were selected for study on the basis of links to a phenotype. The DNMT3B variant has been linked to cancer [21]–[23] and Downs Syndrome [24]. The DNMT3L variant has been linked to reproductive function [25], [26] and intelligence [27]. The DNMT1 and 3A SNPs were selected on the basis of our own unpublished observations in relation to cancer and reproduction. The DNMT3L variant is a nonsynonymous polymorphism resulting in an amino acid change at codon 278 within the C-terminal portion of DNMT3L. Such nonsynonymous variations in the functional domains of the DNMTs are unusual and no non-synonymous polymorphisms have been detected in the catalytic domains of DNMT3A/B or DNMT1 in a European population [28]. Genotyping was carried out by laboratory staff blind to the birth outcomes. All eligible cases in which DNA was available were measured and all valid results included in the analysis. Any incompatible maternal and baby genotypes were excluded. DNMT results were available in 1,236 mother and 1,073 offspring. The smaller number in the offspring reflected the practical difficulty of obtaining cord blood from all recruited pregnancies.

### Methylation

We studied methylation in three imprinted genes and one retrotransposon in genomic DNA extracted from cord blood. Methylation was measured by pyrosequencing using a PyroMark MD system (Qiagen, Crawley, UK) after bisulphite conversion of DNA using Epitect Bisulfite kits (Qiagen, Crawley, UK). Long interspersed nuclear element 1 (LINE-1; OMIM 151626) is a class II retroposon which can be transcribed into RNA, reverse transcribed into cDNA, and then reintegrated as cDNAs into the genome at a new location. The genome retrotransposon content varies between individuals but there are over 500,000 LINE-1 copies in the human genome with around 80–100 active elements [29]. Insulin-like growth factor II (IGF2; 11p15) has been implicated in fetal growth, imprinting syndromes, Wilms Tumour, obesity, metabolic syndrome (OMIM 147470). Paternally expressed gene 3 (PEG3; 19q13) is a regulator of TNF response and has been implicated in tumour development (OMIM 601483). Small nuclear ribonucleoprotein polypeptide N (SNRPN; 15q12) is a bicistronic maternally methylated imprinted gene that encodes 2 polypeptides, the SmN splicing factor, which is involved in RNA processing, and the SNRPN upstream reading frame (SNURF) polypeptide, it is associated with Prader-Willi syndrome and Angelman syndrome, two clinically distinct neurogenetic disorders (OMIM 182279). Assays were carried out using previously published primer sets for IGF2 [30], PEG3 [31], and SNRPN [32] and a commercially available kit for LINE1 (Qiagen, Crawley, UK). The number of CpG sites was 7 for PEG3, and 4 for SNRPN, IGF2 and LINE1. The methylation level at adjacent CpG sites was highly correlated therefore an average methylation level for each gene/region was used in the analysis.

### Statistics

Statistical analysis was carried out using STATA/SE version 12 (Stata Corp, College Station, Texas, USA). Comparison of genotype frequencies in mothers and offspring was tested by a Pearson Chi-square test. Genotypes were also tested for deviation from Hardy-Weinberg equilibrium. Regression analysis was carried out for each variant on the basis of an intermediate heterozygote effect (homozygote minor frequency>heterozygote>homozygote common). Multiple linear regression was used for continuous dependent variables (baby weight, placental weight, baby length, gestational age) and logistic regression for categorical outcomes (low birth weight, pre-term birth, neonatal treatment), with adjustment for appropriate covariates (specified in the results section). Regression results are presented with 95% confidence intervals and p values.

## Results

The mean characteristics of the mothers studied were; age at delivery 30.5 (SD 5.4) years; height 164 (SD 7) cm; gestational age at delivery 39 (SD 2) weeks; weight at booking 69 (SD 15) kg. The birth weight was 3475 (SD 531) g; placental weight 645 (SD 143) g; crown heel length 50 (SD 2) cm; gestation length 39 (SD 2) wks. Births before 37 weeks gestation made up 4.3 (95% CI 3.1,5.4) % of pregnancies and babies requiring neonatal care 15.3 (95% CI 13.3,17.3) %. The lowest birth weight decile, by definition, made up 10% of cases. The number of non-Caucasian mothers was small (n = 38). 15 (95% CI 13, 17) % of the mothers were smokers.

The distribution of genotypes was not significantly different between mothers and babies (**Table 1**) and there was no evidence of significant deviation from Hardy-Weinberg equilibrium. Birth weight, adjusted for gestational age and sex, was significantly related to the baby DNMT3L genotype (**Table 2**). The minor allele was associated with a dose dependent increase in adjusted birth weight of 54 (95% CI 10,99) g (p = 0.016). The risk of being in the lowest decile for standardised birth weight (adjusted for gestation and baby sex) was also related to the baby DNMT3L allele (odds ratio = 0.63; 95% CI = 0.42,0.92; p = 0.018). The baby DNMT3L allele was associated with birth length 0.23 (95% CI 0.04,0.42) cm (p = 0.017) and placental weight 18 (95% CI 3,33) g (p = 0.017) after adjustment for gestational age and baby sex.

**Table 1:** DNMT genotypes and allele frequencies in mothers and babies.

	Gene (variant)			
	*DNMT1* (rs2162560)	*DNMT3A* (rs734693)	*DNMT3B* (rs2424913)	*DNMT3L* (rs7354779)
**Genotypes**				
Homozygote common: mother	380 (32.1)	634 (52.4)	357 (29.3)	622 (52.3)
baby	336 (33.2)	536 (51.2)	333 (31.3)	532 (52.3)
Heterozygote: mother	593 (50.0)	474 (39.1)	609 (50.0)	465 (39.1)
baby	492 (48.6)	427 (40.8)	512 (48.2)	422 (41.5)
Homozygote minor: mother	212 (17.9)	103 (8.5)	253 (20.8)	103 (8.7)
baby	185 (18.3)	84 (8.0)	218 (20.5)	63 (6.2)
Total: mother	1,185 (100)	1,211 (100)	1,219 (100)	1,190 (100)
baby	1,013 (100)	1,047 (100)	1,063 (100)	1,017 (100)
**Allele frequencies**				
Common allele: mother	0.57	0.72	0.54	0.72
baby	0.57	0.72	0.55	0.73
Minor allele: mother	0.43	0.28	0.46	0.28
baby	0.43	0.28	0.45	0.27

Genotype frequencies are shown with the percentages in brackets. Allele frequencies are expressed as proportions. All genotypes were in Hardy-Weinberg equilibrium (Chi-squared test).

**Table 2:** Continuous birth outcomes and their relationship to DNMT variants in mothers and babies.

	Gene^1^							
	*DNMT1*		*DNMT3A*		*DNMT3B*		*DNMT3L*	
	Coefficient(95% CI)	pvalue	Coefficient(95% CI)	pvalue	Coefficient(95% CI)	pvalue	Coefficient(95% CI)	pvalue
**Maternal genotype**								
Birth weight (g)^2^	3	0.895	−41*	0.042	10	0.580	11	0.590
	(−35, 40)		(−80, −1)		(−26, 46)		(−29, 50)	
Crown heel length (cm)^ 2^	−0.03	0.744	−0.05	0.562	0.04	0.622	0.10	0.227
	(−0.18, 0.13)		(−0.22, 0.12)		(−0.12, 0.19)		(−0.06, 0.27)	
Placental weight (g)^ 2^	1	0.897	−10	0.115	1	0.839	6	0.383
	(−11, 13)		(−23, 2)		(−10, 13)		(−7, 18)	
Gestation at delivery (weeks) ^3^	−0.03	0.652	−0.03	0.724	−0.19**	0.006	0.11	0.128
	(−0.17, 0.11)		(−0.17, 0.12)		(−0.32, −0.05)		(−0.03, 0.26)	
**Baby genotype**								
Birth weight (g)^ 2^	−7	0.736	8	0.699	22	0.237	54*	0.016
	(−46, 32)		(−34, 51)		(−15, 61)		(10, 99)	
Crown heel length (cm)^ 2^	−0.03	0.711	0.06	0.521	0.06	0.439	0.23*	0.017
	(−0.20, 0.14)		(−0.12, 0.24)		(−0.10, 0.22)		(0.04, 0.42)	
Placental weight (g)^ 2^	0.3	0.968	−5	0.482	3	0.599	18*	0.017
	(−12, 13)		(−19, 9)		(−9, 16)		(3, 33)	
Gestation at delivery (weeks) ^3^	0.02	0.786	−0.0007	0.992	−0.14*	0.035	0.01	0.943
	(−0.12, 0.15)		(−0.15, 0.15)		(−0.27, −0.01)		(−0.15, 0.16)	

1 Variants specified in Table 1. Linear regression analysis based on all three genotype frequencies (homozygote minor frequency>heterozygote>homozygote common) adjusted for baby sex, and gestational age ^2^ or baby sex alone ^3^.

* *p*<0.05,

** *p*<0.01,

*** *p*<0.001.

The imprinted genes PEG3 and IGF2 were significantly related to placental size; 6.9 (95% CI 3.6,10.3; p<0.001) g/% change in methylation for PEG3 and −4.2 (95% CI −6.2, −2.2; p<0.001) g/% change in methylation for IGF2 (**Table 3**). There were no significant relationships between gestational age and baby length and methylation in any of the regions studied. There was a borderline significant association between birth weight, adjusted for gestational age and baby sex and methylation of LINE1 (−13.5 95% CI −27.2,0.1; p = 0.052; g/% change in methylation) and IGF2 (−6.1 95% CI −12.2, −0.1; p = 0.052; g/% change in methylation). Any effect on birth weight appears to span the full birth weight range as the risk of being in the lowest birth weight decile was not related to DNA methylation.

**Table 3:** Continuous birth outcomes and their relationship to to LINE1, PEG3, SNRPN and IGF2 methylation in cord blood.

	Methylation status							
	LINE 1		PEG3		SNRPN		IGF2	
	Coefficient(95% CI)	PValue	Coefficient(95% CI)	pvalue	Coefficient(95% CI)	pvalue	Coefficient(95% CI)	pvalue
Birth weight (g)^ 1^	−13.546	0.052	3.032	0.575	1.901	0.602	−6.084	0.052
	(−27.182,0.089)		(−7.562,13.626)		(−5.243,9.045)		(−12.227,0.059)	
Crown heel length (cm)^ 1^	−0.0291	0.326	0.0149	0.512	−0.0048	0.752	−0.0161	0.222
	(−0.0871,0.0289)		(−0.0297,0.0595)		(−0.0348,0.0252)		(−0.0420,0.00976)	
Placental weight (g)^ 1^	−0.684	0.763	6.948***	<0.001	0.526	0.655	−4.225***	<0.001
	(−5.128,3.760)		(3.576,10.319)		(−1.782,2.835)		(−6.218, −2.232)	
Gestation at delivery (weeks) ^2^	−0.00718	0.747	0.0102	0.552	0.0119	0.301	−0.0126	0.208
	(−0.0508,0.0364)		(−0.0235,0.0440)		(−0.0107,0.0346)		(−0.0321,0.0070)	

Linear regression analysis of DNA methylation on birth parameters. Analyses were adjusted for baby sex, and gestational age ^1^ or baby sex alone ^2^.

* *p*<0.05,

** *p*<0.01,

*** *p*<0.001.

The risk of requiring neonatal care after birth was significantly related to gestational age at delivery and baby sex (**Table 4**). After adjustment for gestational age, birth weight, and sex the baby DNMT3L minor frequency allele was associated with a reduced risk of requiring neonatal care (odds ratio = 0.73; 95% CI = 0.54,0.98; p = 0.039). The risk of requiring neonatal treatment was also related to methylation within LINE1 (p = 0.010) and SNRPN (p = 0.001) (**Table 5**).

**Table 4:** Categorical birth outcomes and their relationship to DNMT variants in babies.

	Gene^1^							
	*DNMT1*		*DNMT3A*		*DNMT3B*		*DNMT3L*	
	Odds ratio(95% CI)	pvalue	Odds ratio(95% CI)	pvalue	Odds ratio(95% CI)	pvalue	Coefficient(95% CI)	pvalue
Risk of low birth weight (lowest decile) ^2^	1.19	0.266	0.76	0.125	1.12	0.443	0.63*	0.018
	(0.88, 1.61)		(0.54, 1.08)		(0.84, 1.49)		(0.42, 0.92)	
Risk of need for neonatal treatment ^3^	0.97	0.837	1.07	0.622	0.87	0.279	0.73*	0.039
	(0.76, 1.25)		(0.82, 1.40)		(0.68, 1.12)		(0.54, 0.98)	

1 Variants specified in Table 1. Logistic regression analysis based on all three genotype frequencies (homozygote minor frequency>heterozygote>homozygote common). The standardised birth weight is already adjusted for gestational age and baby sex ^2^; the risk of requiring neonatal treatment was adjusted for gestational age, baby sex and baby weight ^3^.

* *p*<0.05,

** *p*<0.01,

*** *p*<0.001.

**Table 5:** Categorical birth outcomes and their relationship to LINE1, PEG3, SNRPN and IGF2 methylation in cord blood.

	Methylation status							
	LINE 1		PEG3		SNRPN		IGF2	
	Odds ratio(95% CI)	pvalue	Odds ratio(95% CI)	pvalue	Odds ratio(95% CI)	pvalue	Coefficient(95% CI)	pvalue
Risk of low birth weight(lowest decile) ^1^	1.030	0.580	0.951	0.232	1.017	0.484	1.041	0.096
	(0.929,1.141)		(0.875,1.033)		(0.970,1.066)		(0.993,1.092)	
Risk of need for neonatal treatment ^2^	0.893*	0.010	0.939	0.082	0.902**	0.001	0.989	0.566
	(0.818,0.974)		(0.874,1.008)		(0.851,0.957)		(0.951,1.028)	

Logistic regression analysis. The standardised birth weight is already adjusted for gestational age and baby sex ^1^; the risk of requiring neonatal treatment was adjusted for gestational age, baby sex and baby weight ^2^.

* *p*<0.05,

** *p*<0.01,

*** *p*<0.001.

**Figure 1:**
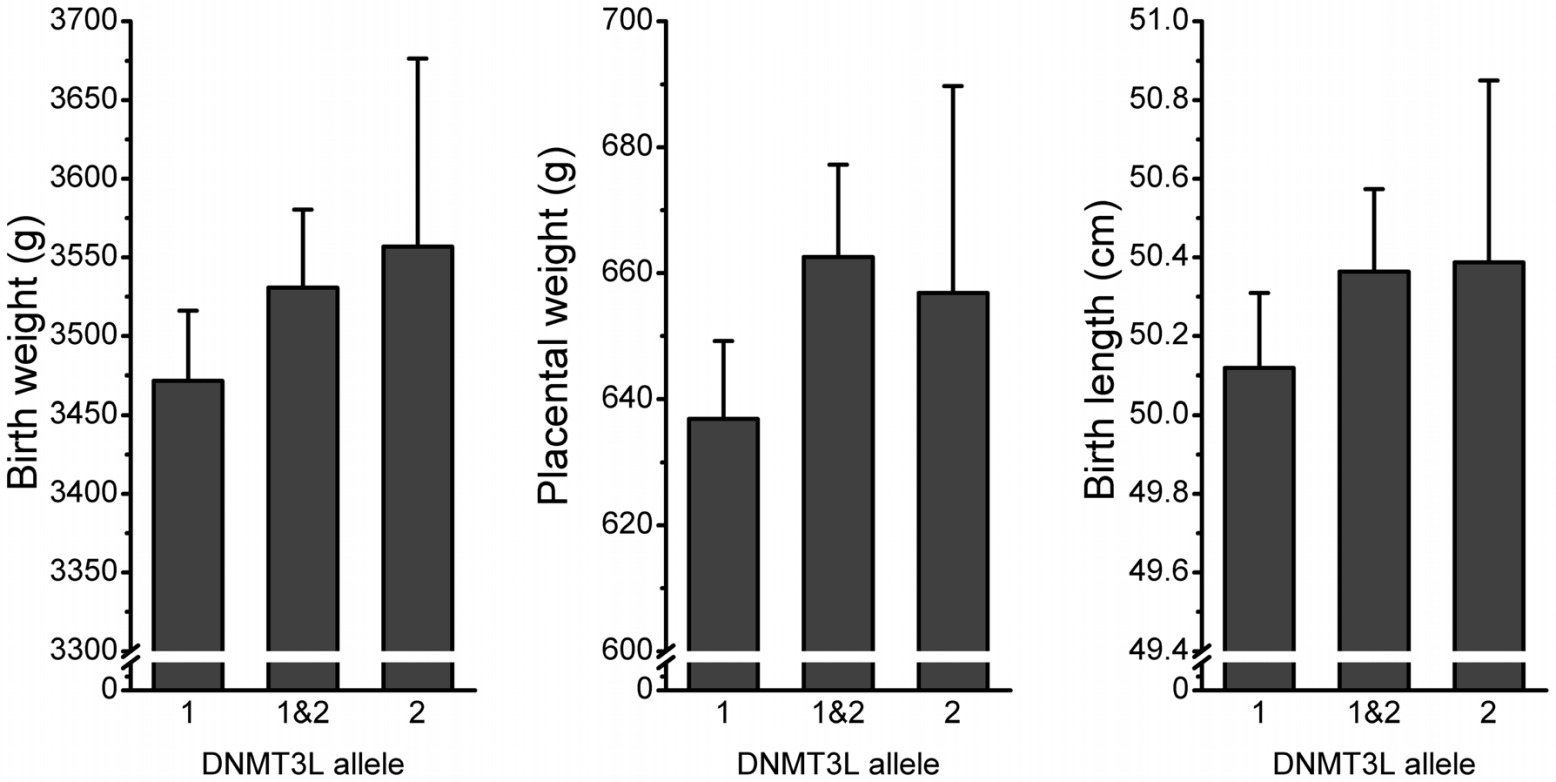
Birth weight, placental weight and birth length by baby DNMT3L genotype. Raw data unadjusted for any other variable. Error bars represent 95% confidence intervals.

**Figure 2:**
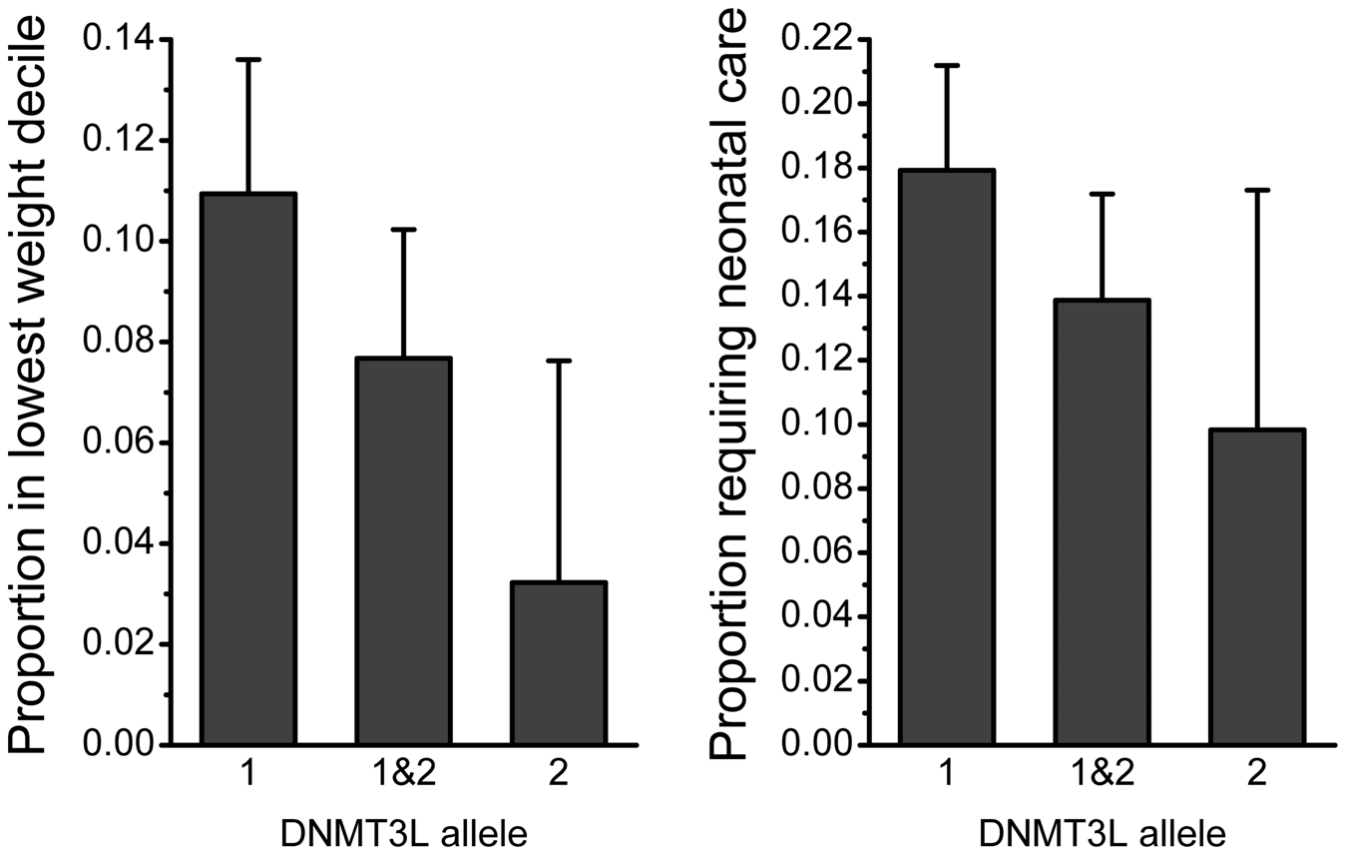
Proportion of babies in lowest standardised birth weight decile and the proportion requiring neonatal treatment by baby DNMT3L genotype. Raw data unadjusted for any other variable. Error bars represent 95% confidence intervals.

For those outcomes significantly related to birth outcomes the unadjusted means are shown by baby DNMT3L allele in **Figures 1** and **2**. Birth weight and birth length both demonstrated intermediate heterozygote effects, though for placental weight the carriers of the minor frequency allele (homozygote and heterozygote) were associated with the similar placental weights (**Figure 1**). Intermediate DNMT3L heterozygote effects were also observed for the proportions of babies in the lowest decile for birth weight and the proportion requiring neonatal care (**Figure 2**). There was no evidence of an effect of maternal DNMT3L genotype on any of the outcomes studied.

There were also no significant relationships between maternal or baby DNMT1, 3A and 3B genotype and birth length or placental weight. However, mother and baby DNMT3B genotype was related to the length of gestation (**Table 2**). The genotype effect was most significant in the mother where the DNMT3B minor frequency allele was associated with a small reduction in gestation length of 1.3 days (p = 0.006) but an almost doubling of the risk of prematurity (<37 weeks gestation); odds ratio = 1.95; 95% CI = 1.30,2.94; (p = 0.001). For all the variants studied there was, as expected, significant covariance between maternal and baby genotype and this appears to explain the baby DNMT3B genotype association with gestation as only the maternal genotype remained significantly associated with gestation when both the maternal and baby DNMT3B genotypes were included in the regression analysis. The most striking and consistent effect of genotype was that between DNMT3L and birth outcome but there was also a separate effect of DNMT3A on birth weight where the minor allele was associated with a lower birth weight (**Table 2**). The effect size was similar to that for DNMT3L (−41 95% CI −80, −1 g) but the level of significance was lower (p = 0.042). There was no evidence of interaction between baby DNMT3L and maternal DNMT3A genotype in relation to birth weight.

All significant associations remained significant after adjustment for smoking. Removal of the non-Caucasian pregnancies made no significant difference to any of the population genotype frequencies and all the significant DNMT3L associations remained significant after exclusion of non-Caucasian mothers from the analysis. The effect of DNMT3A genotype on birth weight and DNMT3B on length of gestation were the least significant before the exclusion and just below significance following the reduction in numbers; though the stronger DNMT3B effect on risk of prematurity remained highly significant (p = 0.002) after exclusion of the non-Caucasian pregnancies.

Simple Bonferonni correction results in borderline significance for the DNMT3L effects but we believe that this adjustment is too conservative. It does not take account of the fact that the genotype was linked to multiple birth outcomes (birth weight, risk of low birth weight, birth length, placental weight, risk of requiring neonatal care). Nor does it take into account the additional biological plausibility conferred by the observation of an intermediate heterozygote effect for all but one of these outcomes.

There were also significant relationships between DNMT genotype and level of methylation in cord blood DNA even after exclusion of non-Caucasians. In the full additive model the baby genotype for DNMT3A was related to PEG3 methylation (0.4; 95% CI = 0.1,0.7% methylation; p = 0.012) and the baby 3B genotype to LINE1 methylation (−0.2; 95% CI = −0.4, −0.01% methylation; p = 0.044). The maternal DNMT3L genotype was also related to IGF2 methylation in the cord blood but this effect was only seen in carriers of the minor frequency allele compared to non-carriers (−0.7; 95% CI = −1.3, −0.0001% methylation; p = 0.050).

## Discussion

Babies carrying the DNMT3L minor allele were heavier at birth. They were also longer and their placentas were heavier. These associations remained significant after adjustment for gestational age and sex. Compared to babies homozygous for the common allele, those homozygous for the minor frequency allele were a third as likely to be in the lowest decile for birth weight and there was a clear allele dose response (homozygote minor frequency>heterozygote>homozygote common). An intermediate effect of the heterozygote is not required to establish a link between a gene variant and health outcome but such an effect provides additional assurance that the association is likely to be biologically significant. There was also an association between maternal DNMT3A genotype and birth weight but the significance level was lower than for DNMT3L and there was no corresponding effect on birth length or placental weight. The DNMT3B variant in the mother was associated with a small effect on the average length of gestation but the effect on prematurity (birth before 37 weeks gestation) was more substantial, with the minor frequency allele being associated with an approximate doubling of the risk of prematurity.

The most striking effect was observed in relation to the DNMT3L variant in the baby. The fact that the association was observed for both birth weight and length suggests that the genotype is linked to body scaling rather than a specific body component such as fat mass. The genetic effect on birth weight was relatively large, with each addition of the 3L minor frequency allele accounting for an additional 54 g in birth weight. This is similar in magnitude to the influence on birth weight of environmental tobacco smoke during pregnancy [33]. However, the DNMT3L association is unlikely to be of value in the clinical management of pregnancy, not least because the predictive genotype resides in the baby’s DNA which is not easy to sample safely. The main value of this finding is that it points to specific biological processes which are worthy of further study in relation to birth outcome, and possibly also longer term health and biological function.

The significant effects reported here were all observed in the DNMT3 family of methyltransferases. The DNMT3L variant results in an amino acid change at codon 278 but the effect of this change has yet to be elucidated. DNMT3A, 3B and 3L are central to the process of de novo methylation [34]–[36] and histone modification through interaction with histone deacetylase [37], [38]. The DNMT3 family is also essential for imprinting [35], [39]. All are expressed in oocytes coincident with imprinting methylation [40] and Dnmt3L is highly expressed in the chorion which contains the trophoblast stem cells [41]. Animal knockouts of the Dnmt3 family result in loss of imprinting, biallelic expression of imprinted genes, and altered methylation at non imprinted loci [34]–[36] but there are species differences in epigenetic regulation and the functional effects of the DNMT3 family in humans are not fully understood. One way to address this in humans is to look at the effect on methylation of genotype within these genes. In this cohort baby DNMT3A genotype was related to PEG3 methylation and DNMT3B genotype to LINE1 methylation. DNMT3L genotype was also related to IGF2 methylation. However, in this case it was the maternal genotype that was important, possibly indicating that this variant has its effect at an earlier stage of development. The timing of imprinting in relation to development varies depending on the region [42].

Imprinted genes have a central role in controlling the fetal demand for nutrients and the placental supply and IGF2 in particular is thought to be important in modulating fetal nutrient transport [43]. In this cohort offspring IGF2 and PEG3 methylation were strongly related to placental weight and IGF2 was borderline significantly associated with birth weight. Transposable elements that flank imprinted genes are thought to be important in setting and maintaining the imprint [44], [45] and they also demonstrate parent-of-origin effects characteristic of imprinting [46]. Transposable elements such as LINE1 have the ability to move around the genome and cause abnormal function and disease if inserted into an important conserved sequence [29], [47], and this process is inhibited by methylation [29]. There is a report of a biphasic relationship between LINE1 methylation and birth weight in a smaller study [48]. This was not observed here though we did observe a linear association between birth weight and LINE1 methylation at p = 0.052.

There was evidence that epigenetic processes were associated with general health in the offspring at the start of life, independently of the links to birth weight and placental weight. DNMT3L genotype in the baby and methylation within LINE1 and the imprinted gene SNRPN were linked to the health of the newborn (risk of requiring neonatal treatment) after adjustment for birth weight, sex and gestational age.

In terms of later health, loss of imprinting, of IGF2 in particular, is a common characteristic of many cancer types including; breast, lung, colon, liver, ovary but it has been proposed that more subtle epigenetic changes could play a seminal role in the earliest steps in cancer initiation [49]–[51] and variation in imprinting occurs in normal tissue of cancer patients or those at increased risk of the disease [49]. The risk of breast cancer increases in a graded way with increasing birth weight [4]. Lower levels of IGF2 methylation have been related to increased cancer risk [52] and IGF2 methylation in this study was associated with a significantly lower placental weight and a borderline significant reduction in birth weight. Fetal growth and brain function are the two main health outcomes associated with the process of imprinting [53]–[55]. Birth weight is positively associated with intelligence [6], [7] and the same minor DNMT3L allele linked here to higher birth weight has also been associated with higher childhood intelligence [27].

The pathways of causality underpinning the epidemiological observations linking early life events and birth outcome to health and biological function throughout life are not well understood. The results here suggest that epigenetic processes are linked to birth outcome and health in early life. The epigenetic links to birth outcome are consistent with some of the known epigenetic associations with later health and biological function. Our emerging understanding of the role of epigenetics in health and biological function across the lifecourse suggests that early epigenetic events could have longer term implications.
